# A direct comparison of the sensitivity of CT and MR cardiac perfusion using a myocardial perfusion phantom

**DOI:** 10.1016/j.jcct.2013.01.016

**Published:** 2013-03

**Authors:** James Otton, Geraint Morton, Andreas Schuster, Boris Bigalke, Riccardo Marano, Luca Olivotti, Eike Nagel, Amedeo Chiribiri

**Affiliations:** a King's College London, Division of Imaging Sciences, The Rayne Institute, 4th Floor Lambeth Wing, St. Thomas' Hospital, London SE1 7EH, United Kingdom; b University of New South Wales, Sydney, Australia; c Department of Cardiology and Pulmonology, Georg-August-University and German Center for Cardiovascular Research, Göttingen, Germany; d Medizinische Klinik III, Kardiologie und Kreislauferkrankungen, Eberhard-Karls-University, Tuebingen, Germany

**Keywords:** Cardiac magnetic resonance perfusion, Cardiac CT perfusion, Phantom, Cardiac computed tomography, Myocardial perfusion imaging, Myocardial CT perfusion, Cardiac MRI

## Abstract

**Background:**

Direct comparison of CT and magnetic resonance (MR) perfusion techniques has been limited and in vivo assessment is affected by physiological variability, timing of image acquisition, and parameter selection.

**Objective:**

We precisely compared high-resolution k-t SENSE MR cardiac perfusion at 3 T with single-phase CT perfusion (CTP) under identical imaging conditions.

**Methods:**

We used a customized MR imaging and CT compatible dynamic myocardial perfusion phantom to represent the human circulation. CT perfusion studies were performed with a Philips iCT (256 slice) CT, with isotropic resolution of 0.6 mm^3^. MR perfusion was performed with k-t SENSE acceleration at 3 T and spatial resolution of 1.2 × 1.2 × 10 mm. The image contrast between normal and underperfused myocardial compartments was quantified at various perfusion and photon energy settings. Noise estimates were based on published clinical data.

**Results:**

Contrast by CTP highly depends on photon energy and also timing of imaging within the myocardial perfusion upslope. For an identical myocardial perfusion deficit, the native image contrast-to-noise ratio (CNR) generated by CT and MR are similar. If slice averaging is used, the CNR of a perfusion deficit is expected to be greater for CTP than MR perfusion (MRP). Perfect timing during single time point CTP imaging is difficult to achieve, and CNR by CT decreases by 24%–31% two seconds from the optimal imaging time point. Although single-phase CT perfusion offers higher spatial resolution, MRP allows multiple time point sampling and quantitative analysis.

**Conclusion:**

The ability of CTP and current optimal MRP techniques to detect simulated myocardial perfusion deficits is similar.

## 1 Introduction

Myocardial perfusion is a major determinant of cardiovascular risk and is an essential tool for the guidance of interventional strategies.^1^ Magnetic resonance perfusion (MRP) represents a highly accurate clinical perfusion imaging technology,^2^, ^3^ with higher spatial resolution than single-photon emission CT^4^ and excellent correlation with invasive fractional flow reserve (FFR) data.^5^

The potential use of CT for the assessment of myocardial perfusion has long been recognized^6^; however, only recently has the advent of fast multislice CT technology resulted in potential widespread clinical application. The most prevalent method of CT perfusion (CTP) is a single time point comparison of myocardial contrast densities at rest and pharmacologic stress. A major multicenter trial of this CTP methodology^7^ has recently concluded.

Although CTP findings correlate well with MRP,^8^, ^9^, ^10^ direct and precise comparison of the sensitivity of the 2 techniques is hampered by several factors, including the lack of an adequate noninvasive “gold standard,” the wide variety of acquisition modes of both MRP and CTP, and physiological and disease variability. Although data from animal models have been useful for the validation of both MRP^11^, ^12^, ^13^ and CTP^14^, ^15^ individually, prolonged anesthesia and contrast accumulation make this technique problematic for systematic side-by-side comparison of multiple perfusion modes.

We therefore used a validated myocardial perfusion phantom^16^ to precisely compare high-resolution k-t SENSE MRP at 3 T, an optimal available clinical standard, with single-phase CTP under identical perfusion conditions. The comparative sensitivity of each method was evaluated with a variety of simulated perfusion deficits and CT energy levels.

## 2 Methods

### 2.1 Perfusion phantom

**Figure 1:**
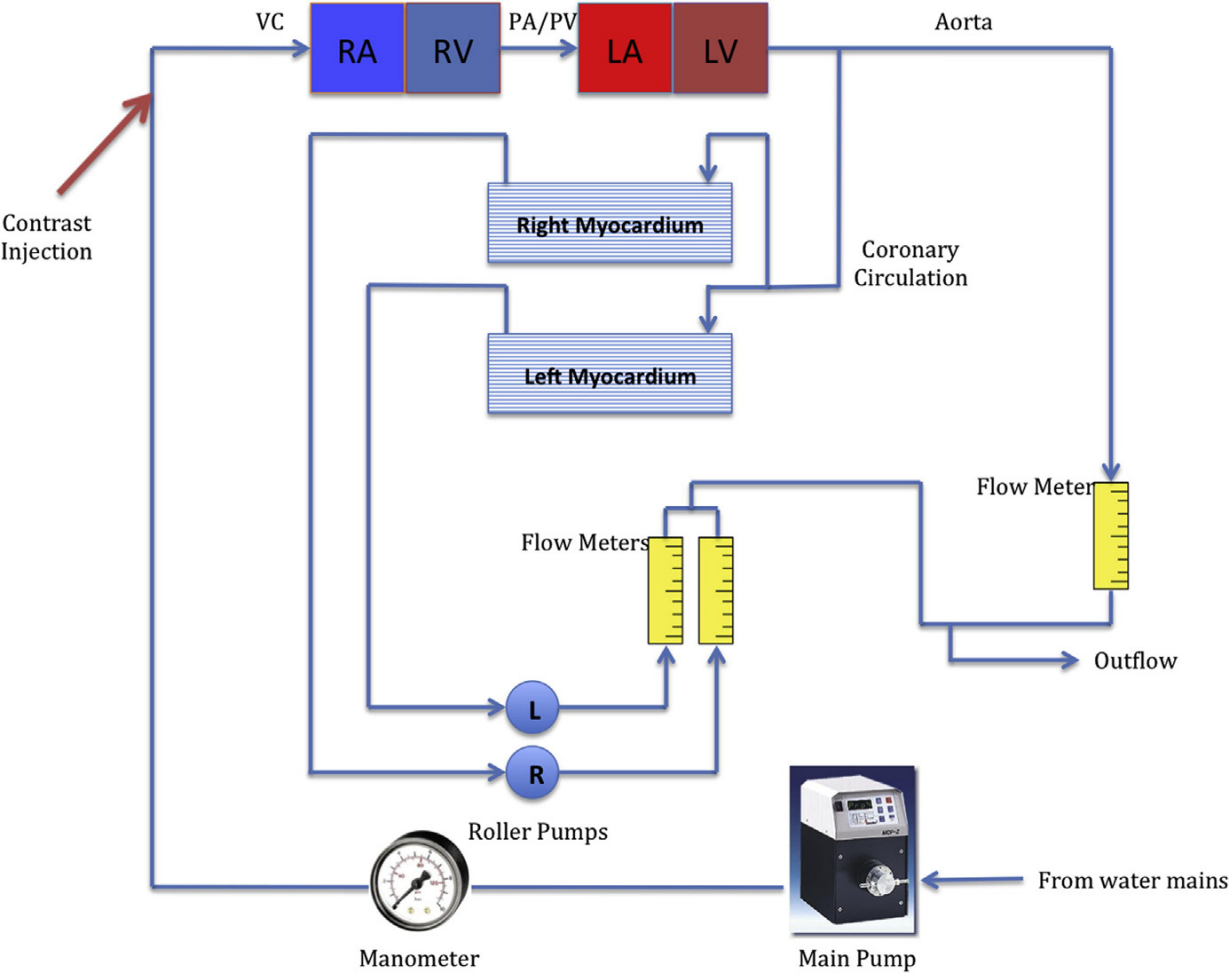
Myocardial perfusion phantom schematic. L, left; LA, left atrium; LV, left ventricle; PA, pulmonary artery; PV, pulmonary vein; R, right; RA, right atrium; RV, right ventricle; VC, vena cava.

Adapted from Chiribiri et al^16^ with permission of Wiley Publishers.

**Figure 2:**
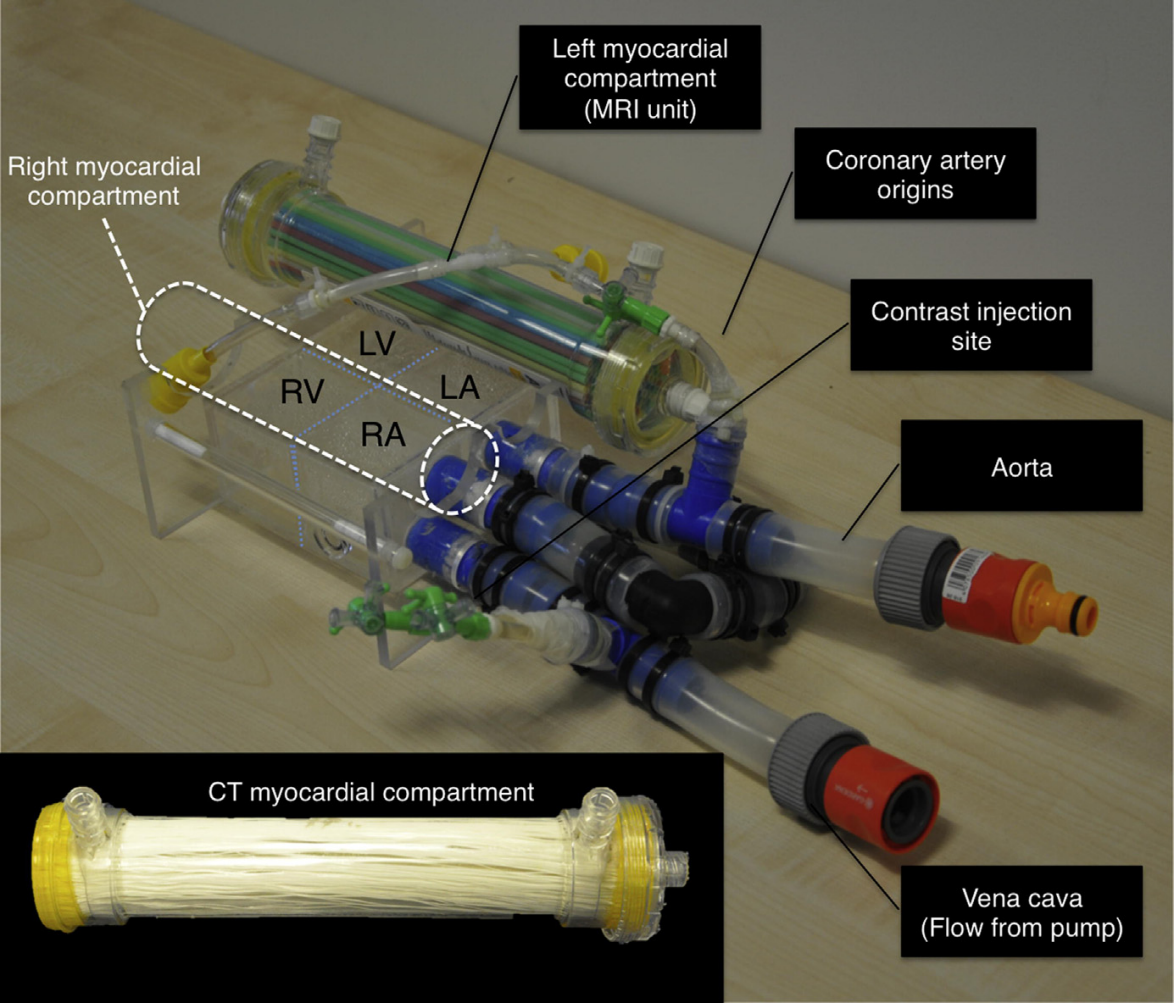
Photograph of the perfusion phantom with magnetic resonance–simulated myocardial compartment. (**Inset**) CT-simulated myocardial compartment. LA, left atrium; LV, left ventricle; RA, right atrium; RV, right ventricle.

Adapted from Chiribiri et al^16^ with permission of Wiley Publishers.

A more detailed description and evaluation of the myocardial perfusion phantom for MRP have previously been published.^16^ A simplified model of the human cardiovascular circulation was constructed, consisting of tubing and mixing chambers to represent the human circulation and to allow physiological contrast dispersion within the model. The phantom includes a venous input, atrial and ventricular cardiac chambers, pulmonary and aortic outputs, coronary arteries, and 2 diffusion chambers to represent myocardial tissue (Figs. 1 and 2).

Input ports on the venous side of the model allow for contrast injection, and coronary arteries that lead from the aortic tubing to 2 chambers connect to myocardial compartments. Flow to the unit was generated with an adjustable continuous flow pump, and phantom outflow and coronary flow were verified with control unit flow meters. A non-recirculating model of circulation was used, adequate to assess first-pass myocardial perfusion. The phantom vascular and chamber volumes are proportional to a small (50–60 kg) adult.

The myocardial compartments consisted of modified hemodialysis filters (AV600; Frezenius SE, Bad Homburg, Germany). In magnetic resonance imaging (MRI) perfusion experiments the polysuflone dialysis fibers were replaced with fine (1.5-mm radius) polypropelene straws to enable contrast diffusion without gadolinium chelate trapping. In the CT experiments the dialysis fibers were retained in situ to allow contrast diffusion while allowing separate composition of the dialysate chamber (Fig. 2, inset). For CT experiments the dialysate chamber was filled with 10% calcium chloride solution to enable a broad spectrum photon absorption, resulting in approximately 30 HU at 120 kV, at the lower normal range of native myocardium before the addition of contrast.^17^ For each experiment 1 myocardial chamber received unmodified flow and served as a control for the ischemic compartment.

### 2.2 MR acquisition methods

MRP was performed at a 3 T Philips Achieva TX system equipped with a 32-channel cardiac phased array receiver coil (Philips, Best, Netherlands). We used a saturation recovery gradient echo method (repetition time/echo time 3.0 milliseconds/1.0 millisecond, flip angle 15°; effective k-t SENSE acceleration 3.8-fold, spatial resolution of 1.2 × 1.2 × 10 mm, saturation-recovery delay of 120 milliseconds). Electrocardiogram (ECG) triggering was simulated at a cardiac frequency of 60 beats/min.

Field strength of 3 T was selected because it provides higher sensitivity than 1.5 T MRI,^18^ and the high-resolution k-t sequence used has been shown to provide superior image quality to standard BTFE imaging^19^ and has been selected for use in a major ongoing MRP clinical trial.^20^ Three-Tesla high spatial resolution k-t accelerated perfusion has shown excellent accuracy in comparison with invasive FFR measurement.^5^ It therefore most likely represents the optimal standard of MRP in current clinical use.

Data were acquired during the first pass of a bolus of 4.5 mL of gadobutrol (Gadovist; Bayer Schering, Leverkusen, Germany) 1 mmol/mL, injected at 4 mL/s, followed by a 20-mL saline flush. CT and MR injection rates and volumes were scaled in proportion with phantom size to replicate clinical aortic contrast curves.

### 2.3 CT methods

CT images where acquired with a Philips iCT 256 detector CT. The perfusion phantom was elevated from the CT gantry while the CT was used in step-and-shoot mode with acquisitions every 1 second. ECG gating at 60 beats/min was simulated with a pacing device. Tube current of 100 mA was used for all experiments with a 0.30-second gantry rotation time. For CT the injection rate was 3 mL/s Iodohexal 370 mg/mL iodine (Ultravist 370) for 10 seconds, corresponding to an iodine delivery rate of 1.11 g/s.

### 2.4 Perfusion image acquisition and analysis

Coronary blood flow to the active chamber was adjusted to 80%, 60%, and 40% of the control chamber corresponding to myocardial perfusion rates of 4, 3, and 2 mL/g per minute, respectively, based on the perfusion volume at the imaging location. Perfusion to the control chamber was maintained at 5 mL/g per minute. Comparative 100-kV and 80-kV acquisitions were also obtained with an 80% myocardial perfusion setting.

Current CTP techniques rely on analysis of contrast inflow into a region of interest (typically the descending aorta) with triggering of the perfusion scan after a short delay. Because the selection of the optimal imaging time point is not possible a priori with current CT methods, both the peak and the average contrast at time points 2 heartbeats before and after peak were evaluated to simulate clinical imaging with minor timing imperfections at various perfusion settings.

All data were analyzed from recorded DICOM data with CT values recorded in Hounsfield units and MR data in arbitrary units of signal intensity. ImageJ version 1.44 (NIH, Bethesda, MD, USA) and ViewForum version 3.1 (Philips Healthcare, Netherlands) was used for Hounsfield and signal intensity measurements within the myocardial chamber. Time was measured from the start of signal upslope for each perfusion setting. Contrast was assessed as the difference between the signal intensity of the underperfused and control compartment. Noise estimates for MRI^19^ and CT^21^ were ascertained from published data, with an expected segmental noise of 20.8 signal units for MRI, and noise values of 18.8, 24.6, and 40.3 for 120-kV, 100-kV, and 80-kV CT, respectively. These estimates agree with our own clinical data.

## 3 Results

**Figure 3:**
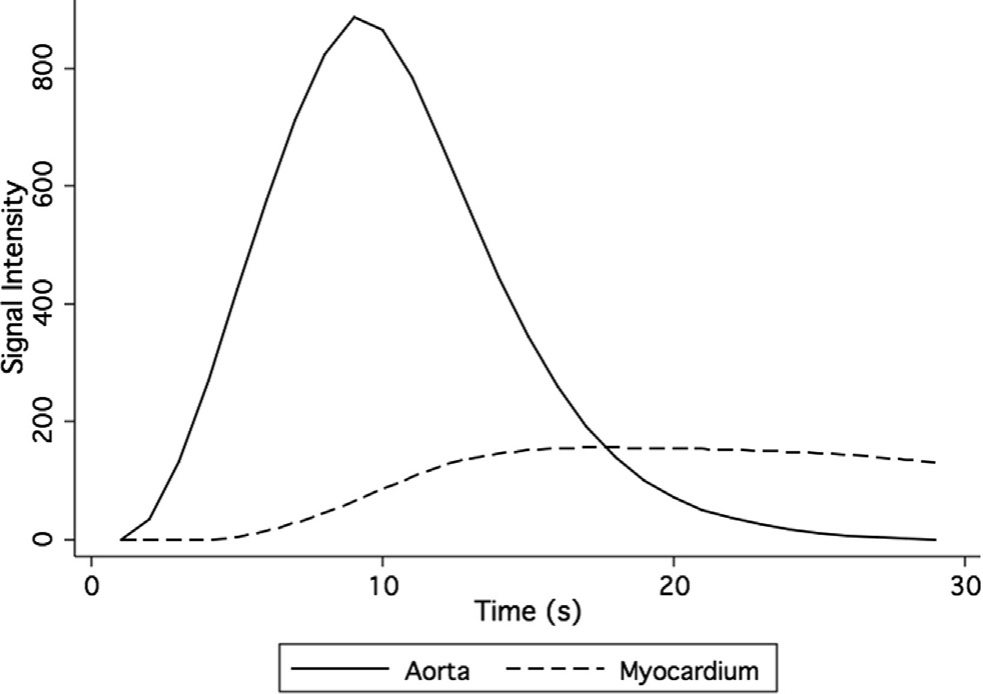
Magnetic resonance imaging phantom perfusion signal intensity.

**Figure 4:**
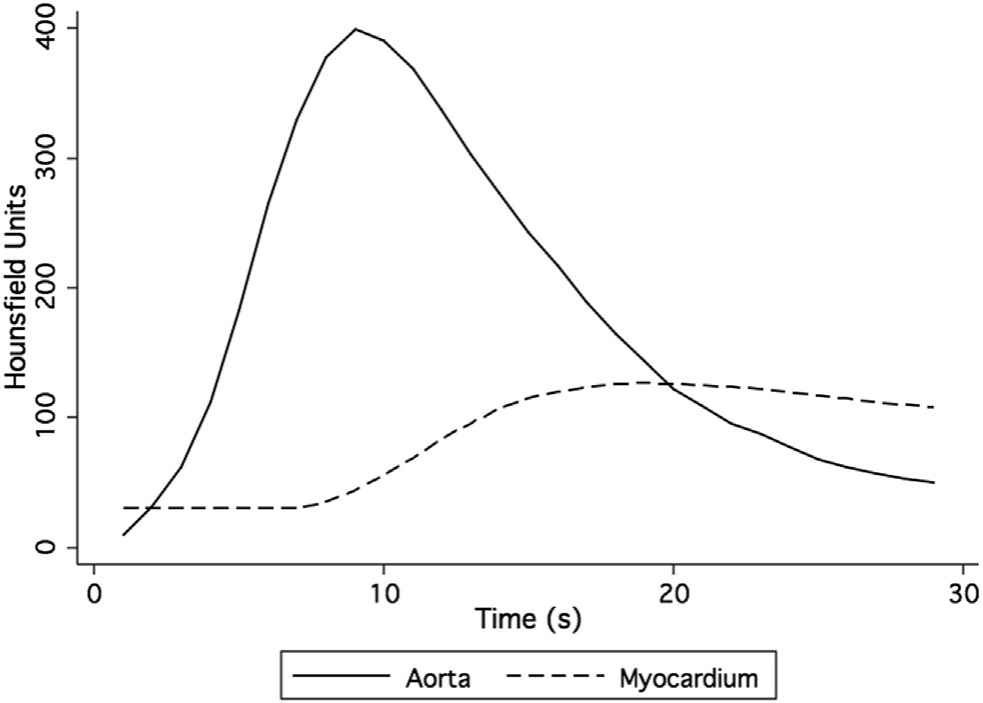
CT myocardial perfusion phantom attenuation.

The aortic contrast density input function and myocardial density functions measured in the phantom resemble clinical and physiological values for both MRI (Fig. 3) and CT (Fig. 4).^22^ Contrast returned to baseline levels with continued flow through the phantom, and no contrast was found to be retained by phantom or simulated myocardium.

### 3.1 CT

**Figure 5:**
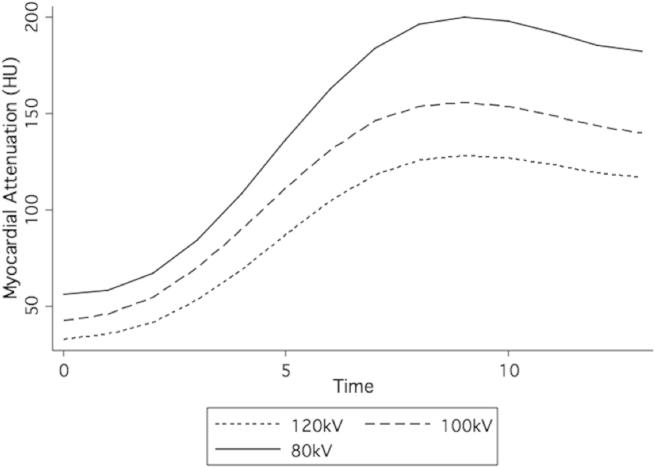
CT myocardial perfusion imaging at various photon energy levels.

**Figure 6:**
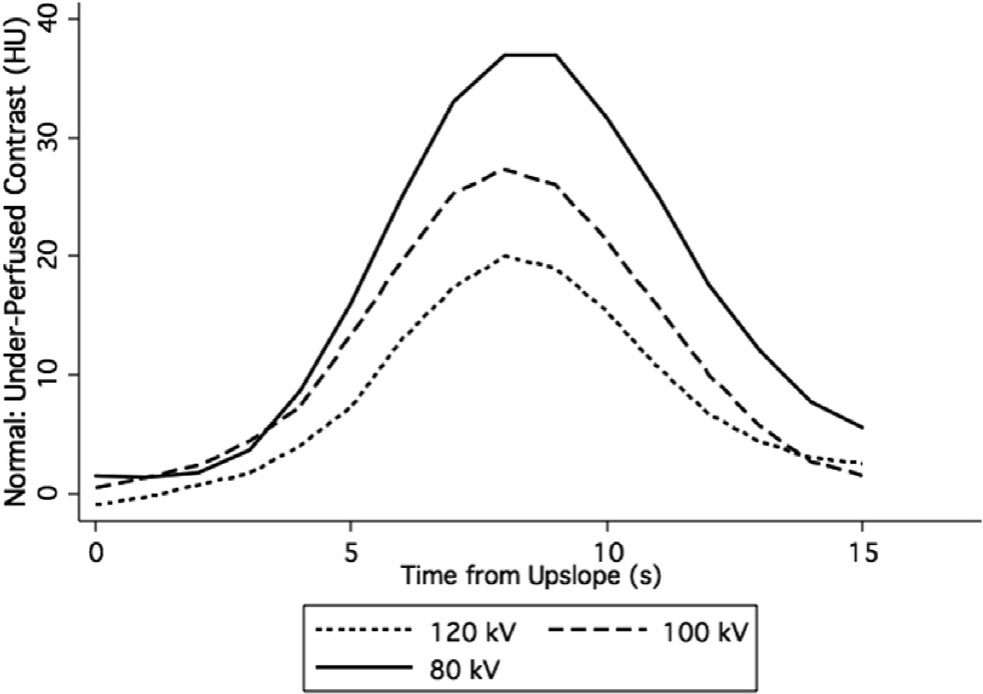
CT myocardial perfusion image contrast between normal and 20% perfusion reduction compartments at varied x-ray photon energy.

**Figure 7:**
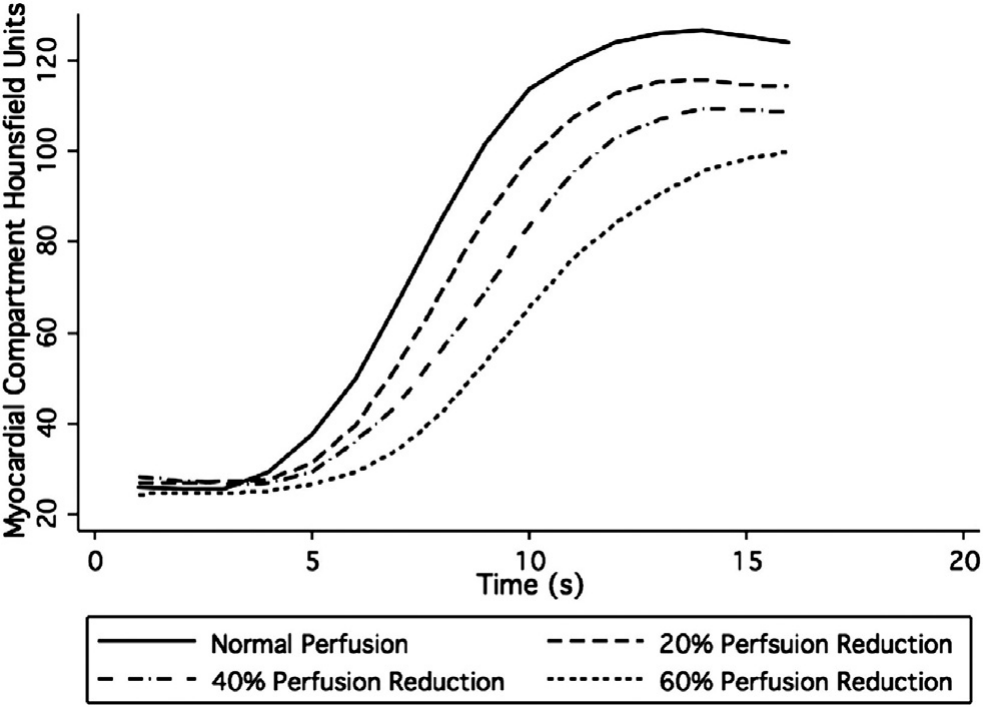
CT myocardial perfusion imaging with simulated perfusion deficits.

Mean Hounsfield unit values and contrast between the normal and underperfused myocardial compartments increased at lower photon energy level values (Fig. 5). The increase in attenuation was approximately commensurate with the increase in noise with lower photon energy level, such that the expected contrast-to-noise ratios (CNRs) are similar (Table 1; Fig. 6). As myocardial perfusion decreases, the contrast between the normal and underperfused compartments increases because of both reduced contrast inflow and delayed contrast upslope (Fig. 7). A 2-second error of timing results in a 24%–31% reduction of contrast between normal and underperfused segments (Table 2).

**Table 1:** Contrast and CNRs for CT and MRI perfusion of a 20% perfusion deficit at various photon energy levels.

	CT 120 kV (CNR)	CT 100 kV (CNR)	CT 80 kV (CNR)	MRI (CNR)
Perfect timing	20 (1.1)	29 (1.2)	40 (1.0)	21 (1.0)
Imperfect timing	15 (0.8)	22 (0.9)	30 (0.74)	—

CNR, contrast-to-noise ratio; MRI, magnetic resonance imaging.

Perfect timing indicates the maximum contrast possible, whereas imperfect timing reflects the average of a 2-second error from the perfect time point.

**Table 2:** Contrast and CNRs for 120-kV CT and MRI perfusion of a 20% perfusion deficit at various perfusion settings.

	80% Contrast perfusion to control (CNR)	60% Contrast perfusion to control (CNR)	40% Contrast perfusion to control (CNR)
CT: perfect timing	20 (1.1)	35 (1.9)	50 (2.7)
CT: imperfect timing	15 (0.8)	24 (1.3)	38 (2.0)
MRI	21 (1.0)	41 (2.0)	62 (3.0)

CNR, contrast-to-noise ratio; MRI, magnetic resonance imaging.

Perfect timing indicates the maximum contrast possible, whereas imperfect timing reflects the average of a 2-second error from the perfect time point.

### 3.2 MRI

High concentrations of gadolinium may lead to saturation effects, and the relationship between gadolinium concentration and the MR signal is nonlinear, particularly at high concentrations as may be found within the left ventricular cavity or aorta. Visual analysis does not show significant saturation effects within the myocardial chamber itself, and myocardial perfusion curves closely resemble those of the corresponding CTP studies (Figs. 3 and 4). As expected, peak contrast between myocardial compartments was contingent on the perfusion deficit but was not linearly related to it.

### 3.3 CT versus MRI CNRs

**Figure 8:**
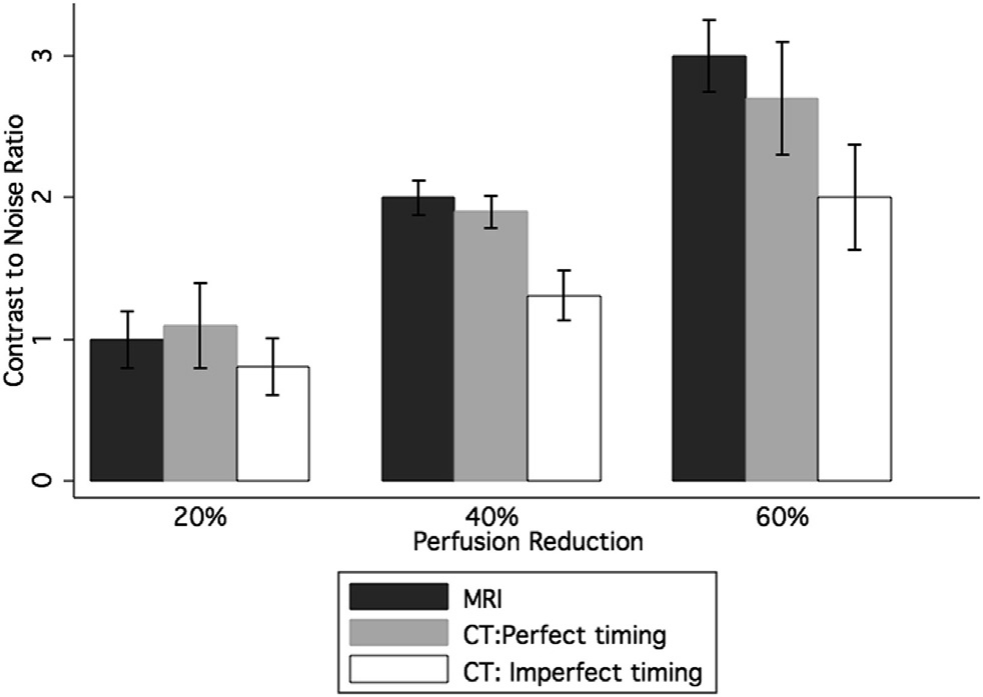
Image contrast for a perfusion deficit of 20% for magnetic resonance perfusion and CTP. CTP contrast both with perfect image acquisition timing and an error of 2 seconds is displayed. 95% Confidence intervals are displayed. CTP, CT perfusion; MRI, magnetic resonance imaging.

Contrast between the perfused and underperfused myocardial chambers and estimated CNR are given in Table 2 and Fig. 8. The CNR for both MRI and CT are similar at all perfusion levels. Imperfect timing of CTP image acquisition (a 2-second timing error) during contrast inflow may lead to a 24%–32% reduction in signal.

The measured CNR reflects the CNR within native images; however, it should be noted that the slice thickness of the MRP sequence used is 10 mm, whereas the CT slice thickness is 0.6 mm, with a smaller voxel volume. Although the effect of slice averaging may not be adequately assessed from the phantom data because of the homogenous nature of the underlying material, from theoretical principles, the CNR for a 10-mm averaged CTP slice would be up to 4 times greater than 0.6-mm slice data. Real-world data suggest an increase in CNR of 45% from thin to 5-mm slice CTP images.^23^ For the same slice thickness, therefore, the CNR for CTP would be expected to be greater than that of MRP.

## 4 Discussion

Despite the entirely distinct physical principles underlying CT and MR image formation and the exquisite sensitivity of proton relaxation to gadolinium-based contrast agents,^24^ our study indicates that the sensitivities of each perfusion modality when directly compared in a phantom model are similar. This finding is important for several reasons.

First, it suggests that, although both CTP and MRP are subject to rapid technologic change, the fundamental properties underlying CTP allow it to be a viable alternative to MRP and supports the further development of the nascent technology.

Second, it reinforces the reliance of current CTP methods on optimal parameter selection. We found a loss of 24%–31% of available image contrast from a relatively small timing error from the ideal sampling point during first-pass perfusion. Timing methods before bolus administration (which would need to be used during vasodilator stress) and careful attention to image acquisition within the late upslope may be required to mitigate this issue. Methods that allow surveillance of aortic contrast density and CT triggering with minimal delay^25^ may limit any loss in the CNR ; however, scanning over multiple heartbeats (eg, axial scanning with 64 or 128 detector rows) will necessarily involve reduction in image contrast at certain levels within the volume.

Attention should also be paid to the photon energy level (as dictated by the kV setting) setting of CTP studies. Given that reducing the photon energy level greatly lowers the effective radiation dose, lower photon energy level may be preferable, at least within the linear portions of the expected noise/body mass index functions.

Third, given the greater resolution of CTP, this research suggests that aggressive CTP dose-reduction strategies, such as reduced photon energy or image undersampling^26^ that trade image quality and voxel size for greatly reduced radiation dose, may nevertheless provide image quality commensurate with that of MRP, while allowing multiple CTP heartbeat acquisitions. This may remove a disadvantage of the most common method of CTP in which only a single heartbeat is acquired. Perfusion kinetics over multiple heartbeats, as captured by MRP, may be useful in detecting deficits and distinguishing imaging artifact,^27^ enabling visual analysis of the changing epicardial-to-endocardial gradients and the duration of periods of localized hypoperfusion.

### 4.1 Limitations

The present research has several limitations. The main benefit of the myocardial perfusion model, namely its reproducibility, is also a weakness because it may not capture the broad range of body structures and physiological states that may be present within the clinical environment. Likewise, the model cannot reproduce the multiple sources of image artifact and noise, including respiration artifact and signal attenuation that affect both CT and MRI. Motion artifact and beam hardening may significantly affect image interpretation but cannot be captured by the current model. The CNRs presented are contingent on published noise estimates, representing an aggregate of clinical data, which may not be applicable to particular circumstances, and do not capture other features such as perfusion gradients that may be of diagnostic use.^27^ Although the factors contributing to image noise, particularly with MRI, are complex, it is likely that the relationship of body habitus and image noise differs between MRI and CT modalities. Our study may therefore have underestimated the relative benefits of MRP in obese patients. New technology such as improved MR coil design or iterative reconstruction of CT^28^ data may alter the relative benefits of one modality.

Although the phantom represents a gross simplification of the cardiovascular system and is incapable of showing myocardial diffusion, it succeeds in its aim of providing realistic aortic contrast intensity functions. Nevertheless, the experiments were performed with a selected injection rate, contrast composition, cardiac output, and imaging devices, and the relative sensitivity of the 2 techniques may be affected by particular adjustments of these parameters. It should also be noted that the simulated myocardium for each modality was slightly different, with larger diameter fibers used in the MR experiments to prevent contrast accumulation.

It should also be noted that both perfusion techniques are subject to rapid technologic change and multiple modes of image processing and analysis. Methods of perfusion quantification from MRI^29^, ^30^, ^31^ have been implemented, and dynamic CT perfusion^8^, ^22^, ^32^ is also in development. We have analyzed a method of visual contrast analysis that reflects current technology and clinical practice; however, the perfusion phantom may be useful in the future for assessing the mathematical models and methods involved in perfusion quantification.

Despite these weaknesses, the overall conclusion for the general comparability of the 2 perfusion techniques appears robust. Previous clinical studies that compared CTP and MRP have indicated that CT has a generally good accuracy when MRP is used as the reference standard.^8^, ^9^ These studies include a broad range of coronary disease, and a perfect arbitrator between the 2 techniques in the case of disagreement does not exist. Nonclinical studies within a controlled environment are therefore important for the assessment of differences between perfusion techniques, particularly in the setting of small or subtle perfusion deficits or when quantitative measurement is required. Future CTP methods, including dual-energy acquisition, novel image processing techniques, and the validation of new methods of quantitative perfusion assessment in both CT and MR environments, may also be assessed with a perfusion phantom technique.

## 5 Conclusion

CTP at least equals the ability of current optimal MRP techniques to detect simulated myocardial perfusion deficits. CTP allows higher spatial resolution and the possibility of slice-averaging techniques for image noise reduction. MRP has the benefit of allowing analysis of contrast inflow dynamics. Both techniques are subject to rapid technologic change, which may overcome the current limitations of both techniques.
